# Anti-inflammatory and immunomodulatory mechanisms of atorvastatin in a murine model of traumatic brain injury

**DOI:** 10.1186/s12974-017-0934-2

**Published:** 2017-08-23

**Authors:** Xin Xu, Weiwei Gao, Shiqi Cheng, Dongpei Yin, Fei Li, Yingang Wu, Dongdong Sun, Shuai Zhou, Dong Wang, Yongqiang Zhang, Rongcai Jiang, Jianning Zhang

**Affiliations:** 1 Department of Neurosurgery, Tianjin Medical University General Hospital, 154 Anshan Road, Tianjin, China; 2 Key Laboratory of Post-Trauma Neuro-Repair and Regeneration in Central Nervous System, Ministry of Education, 154 Anshan Road, Tianjin, China; 3 Department of Neurology, Tianjin Huan Hu Hospital, 6 Jizhao Road, Tianjin, China; 4 Department of Neurosurgery, The Second Affiliated Hospital of Nanchang University, 1 Minde Road, Nanchang, Jiangxi China; 5 Department of Geriatrics, Tianjin Medical University General Hospital, 154 Anshan Road, Tianjin, China

**Keywords:** Traumatic brain injury, Atorvastatin, Immunomodulation, Anti-inflammation, Leukocyte, Microglia/macrophage subtype

## Abstract

**Background:**

Neuroinflammation is an important secondary injury mechanism that has dual beneficial and detrimental roles in the pathophysiology of traumatic brain injury (TBI). Compelling data indicate that statins, a group of lipid-lowering drugs, also have extensive immunomodulatory and anti-inflammatory properties. Among statins, atorvastatin has been demonstrated as a neuroprotective agent in experimental TBI; however, there is a lack of evidence regarding its effects on neuroinflammation during the acute phase of TBI. The current study aimed to evaluate the effects of atorvastatin therapy on modulating the immune reaction, and to explore the possible involvement of peripheral leukocyte invasion and microglia/macrophage polarization in the acute period post-TBI.

**Methods:**

C57BL/6 mice were subjected to TBI using a controlled cortical impact (CCI) device. Either atorvastatin or vehicle saline was administered orally starting 1 h post-TBI for three consecutive days. Short-term neurological deficits were evaluated using the modified neurological severity score (mNSS) and Rota-rod. Brain-invading leukocyte subpopulations were analyzed by flow cytometry and immunohistochemistry. Pro- and anti-inflammatory cytokines and chemokines were examined using enzyme-linked immunosorbent assay (ELISA). Markers of classically activated (M1) and alternatively activated (M2) microglia/macrophages were then determined by quantitative real-time PCR (qRT-PCR) and flow cytometry. Neuronal apoptosis was identified by double staining of terminal deoxynucleotidyl transferase-dUTP nick end labeling (TUNEL) staining and immunofluorescence labeling for neuronal nuclei (NeuN).

**Results:**

Acute treatment with atorvastatin at doses of 1 mg/kg/day significantly reduced neuronal apoptosis and improved behavioral deficits. Invasions of T cells, neutrophils and natural killer (NK) cells were attenuated profoundly after atorvastatin therapy, as was the production of pro-inflammatory cytokines (IFN-γ and IL-6) and chemokines (RANTES and IP-10). Notably, atorvastatin treatment significantly increased the proportion of regulatory T cells (Tregs) in both the peripheral spleen and brain, and at the same time, increased their main effector cytokines IL-10 and TGF-β1. We also found that atorvastatin significantly attenuated total microglia/macrophage activation but augmented the M2/M1 ratio by both inhibiting M1 polarization and enhancing M2 polarization.

**Conclusions:**

Our data demonstrated that acute atorvastatin administration could modulate post-TBI neuroinflammation effectively, via a mechanism that involves altering peripheral leukocyte invasion and the alternative polarization of microglia/macrophages.

## Background

Traumatic brain injury (TBI) is one of the leading causes of mortality and morbidity worldwide, especially in children and young adults [1]. The pathogenesis of TBI is highly complex, involving primary and secondary injury. Immediately following the mechanical stress (primary injury), several cellular and biochemical pathological events (secondary injury), including oxidative stress, inflammation, mitochondrial dysfunction, and apoptosis occur within minutes and may last from hours to days or months [2]. Among these pathological changes, neuroinflammation has been implicated as an important secondary injury mechanism that exerts either detrimental or beneficial roles in the process of TBI-induced central nervous system (CNS) damage [3]. CNS inflammation is a robust, sterile immune reaction that is characterized by CNS-resident glial activation, peripheral immune cells recruitment, and production of cytokines and chemokines [4, 5]. Although balanced inflammation is essential for the disposal of cellular debris and tissue remodeling, sustained and excessive inflammation can exacerbate neuronal apoptosis and neurological impairment after TBI [6, 7].

Massive invasion of circulating immune cells within the CNS has been reported in both TBI patients and animal models [8]. Neutrophils first migrate into the injured brain within 24 h post-injury. Macrophages, T cells, natural killer (NK) cells, and dendritic cells predominate at days 3–5 post-injury [9]. These activated cells secrete pro-inflammatory and cytotoxic mediators, such as reactive oxygen species (ROS) and pro-inflammatory cytokines, thus contributing to neuronal damage [10]. Blood-derived leukocyte recruitment is facilitated through the peri-contusional region or the damaged blood–brain barrier (BBB) [6]. In addition, chemokines produced by activated glia and neurons are also instrumental in leukocyte trafficking [11]. In the acute period after TBI, attenuating peripheral immune cells invasion into the injured CNS exhibited protection against neuroinflammation and secondary neuronal injury [12–14]. In addition to these detrimental events, a recent study indicated that regulatory T cells (Tregs), an immunosuppressive T cell subset, had neuroprotective functions in ameliorating excessive pro-inflammatory responses and tissue damage after TBI [15].

Microglia/macrophages are among the first responders to CNS injuries. After TBI, brain resident microglia and infiltrating macrophages are rapidly activated and undergo marked changes in cell morphology and behavior [16]. Like macrophages in non-CNS tissues, studies now agree that microglia are also highly plastic cells that can be polarized into the “classically activated” M1-phenotype or the “alternatively activated” M2-phenotype, according to their host tissue microenvironments [17, 18]. M1 polarization is involved in pro-inflammatory responses that aggravates tissue damage, which is induced by exposure to microbial products or pro-inflammatory cytokines. In contrast, M2 polarization is associated with the induction of anti-inflammatory cytokines and tends to promote tissue repair [19]. Strategies altering the M2/M1 ratio already have promising effects in variety of brain injury including TBI [17, 20]. Therefore, immunomodulatory approaches targeting the control of peripheral leukocyte invasion and the polarization status of microglia/macrophages might have therapeutic implications.

Statins, 3-hydroxy-3-methyl-glutaryl-coenzyme A (HMG-CoA) reductase inhibitors, are prescribed widely to reduce serum cholesterol levels and lower the risk of cardiovascular events [21]. Accumulating experimental evidence suggests that statins also have potent anti-inflammatory and immunomodulatory properties. For instance, statins can alter the function of both T cells and antigen presenting cells (APC), and inhibit immune cell invasion via modulating the expression of cell adhesion molecules [22, 23]. Recent studies demonstrated another immunomodulatory effect of statins in modulating the suppressive functions and the recruitment of Tregs toward inflammatory tissues [24]. Among statins, atorvastatin has been reported to exert neuroprotective roles in TBI including enhancing angiogenesis, neurogenesis, and reducing neuronal apoptosis [25]. However, whether atorvastatin treatment modulates immune cells at the acute phase of TBI is unknown. Based on its pleiotropic nature, we hypothesized that atorvastatin treatment would reduce peripheral leukocyte recruitment and alter the microglia/macrophage polarization status, thereby ameliorating neuroinflammation and neuronal apoptosis, resulting in improved neurobehavioral outcomes after TBI.

## Methods

### Animals

Male C57BL/6 mice, 6–8 weeks old (20–23 g), were purchased from the Experimental Animal Laboratories of the Academy of Military Medical Sciences (Beijing, China). The experimental protocols for this study were approved by the Tianjin Medical University Animal Ethics Committee (Tianjin, China). All mice were maintained with free access to food and water in a temperature-controlled (20 ± 2 °C) and humidity-controlled (55 ± 5%) vivarium under a 12 h light/dark cycle, and were adapted to the environment for a week before the experiments.

### Experimental design and atorvastatin treatment

**Fig. 1:**
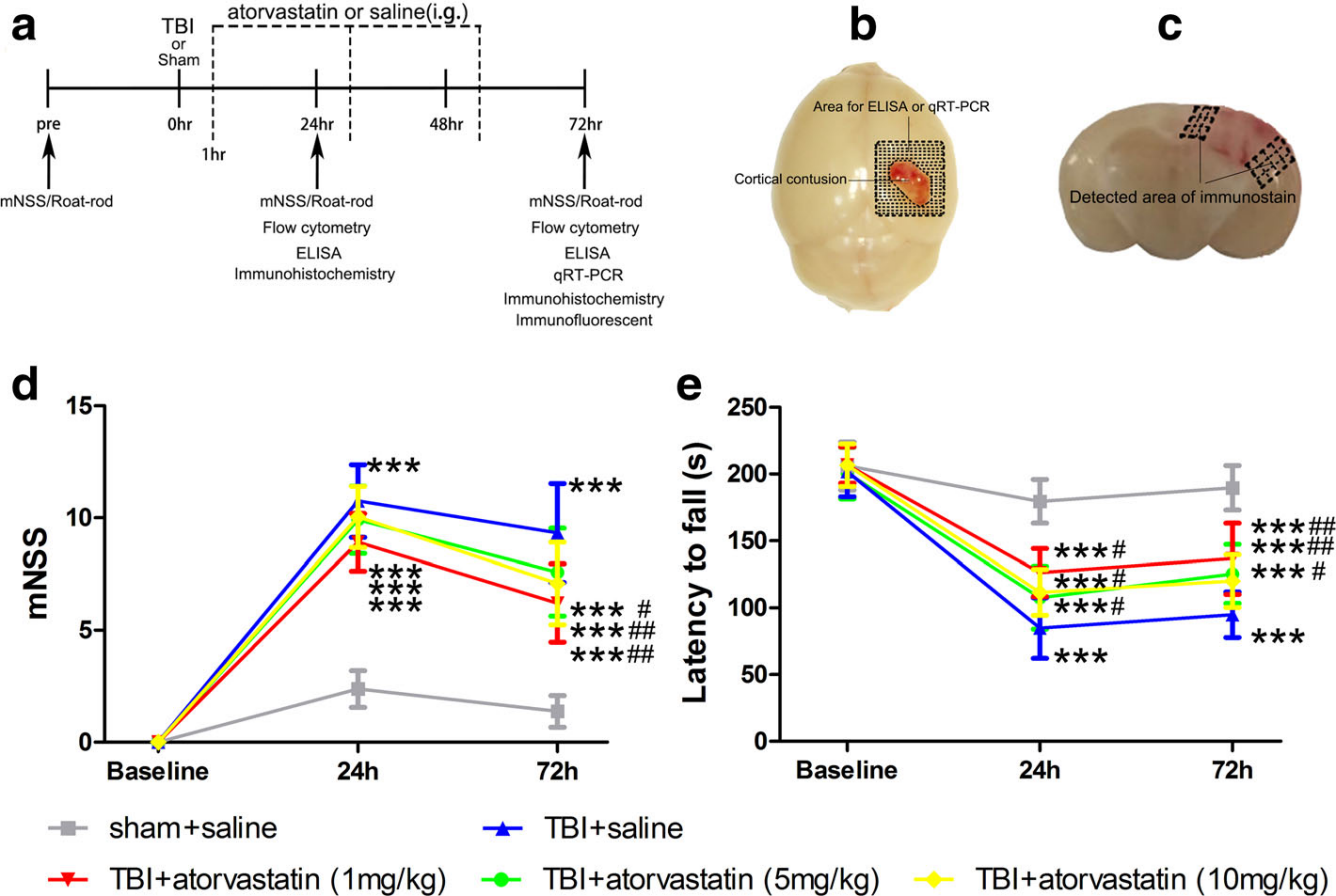
Effect of atorvastatin treatment on neurological outcomes after TBI. **a** A timeline of the experimental design. i.g.: intragastric gavage. **b**, **c** Representative images of the cortical contusion region induced by a CCI system. Shaded areas illustrate the peri-contusional cortex that was harvested for ELISA and qRT-PCR analysis (**b**) and the microphotographed areas used in immunohistochemistry and immunofluorescence (**c**). **d, e** The neurological recovery was analyzed by mNSS (**d**) and Rota-rod(**e**) tests prior to and at 24 and 72 h post-TBI. d mNSS scores were significantly higher in the four TBI groups compared with those in the sham groups. No significant differences were observed among the four TBI groups at 24 h. However, the three different doses of atorvastatin groups showed significantly lower mNSS scores compared with those in the saline group at 72 h post-TBI. No significant differences were detected among the 1, 5, and 10 mg/kg/day atorvastatin groups at any time point. **e** Compared with the mice in the TBI + saline group, atorvastatin (1, 5, and 10 mg/kg/day) administration attenuated the TBI-induced impaired Rota-rod performance at 24 and 72 h after TBI. However, differences in Rota-rod latency among the three atorvastatin groups were not significant on any testing day. Data are presented as the mean ± SD. ***p < 0.001 versus sham group, ^#^ *p* < 0.05 and ^##^ *p* < 0.01 versus TBI + saline group. *n* = 24/group (mNSS) or 12/group (Rota-rod)

A total of 150 mice were randomly assigned to the following four groups: sham + saline group (*n* = 30); sham +1 mg/kg/day atorvastatin group (*n* = 30); TBI + saline group (*n* = 45); TBI + 1 mg/kg/day atorvastatin group (*n* = 45). Post-treatment assessments are shown as a schematic in Fig. 1a. Proper measures were taken to minimize the number of mice used and the pain or discomfort they might experience.

To determine the efficacy of atorvastatin (Atorvastatin calcium tablet, Pfizer Inc.; dissolved in 0.9% saline) on neuroinflammation in the acute phase of TBI, the initial dose was administered orally by intragastric gavage (i.g.) as early as 1 h after TBI [26]. A pilot dose-response analysis was performed using three different doses (1, 5, and 10 mg/kg/day) to determine the optimal dose of atorvastatin, and neurological deficits were evaluated as main outcomes. The results demonstrated that mice treated with atorvastatin performed significantly better than those treated with saline; however, there was no significant difference among the three different doses at all tested time points. Therefore, a dose of 1 mg/kg/day (with the minimal side effect) was chosen for all subsequent experiments. Mice in sham + saline and TBI + saline groups received equal volumes of 0.9% saline with the same schedule.

### Experimental TBI model

TBI was induced using a controlled cortical impact (CCI) device (eCCI-6.3 device, Custom Design & Fabrication, USA). Briefly, mice were anesthetized with 10% chloral hydrate through intraperitoneal (i.p.) injection (3 mL/kg) and then positioned in a stereotaxic frame with a heating pad to maintain body temperature. After the scalp and fascia were retracted, a 3.5-mm diameter hole was drilled on the right cerebral hemisphere (2.0 mm posterior from bregma and 2.0 mm lateral to the sagittal suture) to expose the intact dura. For moderate-TBI induction, a 3-mm-flat impactor tip was used to impact the exposed dual (impact parameters: velocity: 4.5 m/s, depth: 2 mm, dwell time: 200 ms). After CCI injury, the scalp incision was sutured, and the mice were placed in a heating pad until anesthesia recovery. Sham-injured mice received all of these procedures, but did not receive a CCI.

### Tissue preparation

At each time point, mice were anesthetized deeply using 10% chloral hydrate (3 mL/kg, i.p.), the spleens were surgically removed and mononuclear cells were isolated using RBC lysis buffer (eBioscience, San Diego, CA, USA). The anesthetized mice were then perfused intracardially with ice-cold phosphate-buffered saline (PBS). Brains were isolated rapidly and were used as follows. (1) In flow cytometry analysis, brain mononuclear cells were isolated via a discontinous percoll gradient method. Briefly, cerebral hemispheres were gently pressed through a 40-μm nylon cell strainer (BD Bioscience, Franklin Lakes, NJ, USA), the tissue suspension was then resuspended in 5 mL of 30% percoll (Sigma-Aldrich, St Louis, MO, USA), and slowly layered on top of a 5 mL of 70% percoll followed by centrifugation at 500×*g* for 20 min at 18 °C. Mononuclear cells were collected in the 30–70% interphase, washed twice with PBS, and resuspended in 100 μL of flow cytometry staining buffer (eBioscience) for further use. (2) For immunostaining analysis, fresh brains were fixed in 4% paraformaldehyde for 24 h at room temperature and were then embedded in paraffin (for immunohistochemistry) or the Tissue-Tek O.C.T. compound (dehydration by 20% and then 30% sucrose before tissue embedding, for immunofluorescence). Subsequently, a 3-mm thick coronal block, spanning the entire injured cortex, was harvested, and transverse 6-μm thick sections were then cut using a microtome (Leica, Germany). 3) In enzyme-linked immunosorbent assay (ELISA) and quantitative real-time PCR (qRT-PCR) analysis, the peri-contusional cortex (see Fig. 1b) in TBI mice and the equal area in sham-injured mice were dissected on ice, and then immediately frozen in liquid nitrogen. Some of which were homogenized in nine volumes (1:9, *w*/*v*) of ice-cold saline, the supernatants were collected after centrifugation at 1000×*g* for 15 min; the others were immediately immersed in RNA stabilization solution (Invitrogen, Carlsbad, CA, USA) to preserve the RNA quality and quantity.

### Neurobehavioral training and evaluation

Neurological deficits were assessed using well-established modified neurological severity score (mNSS) and Rota-rod at baseline, 24 and 72 h after TBI or sham surgery by two investigators who were blinded to the experimental design. Each behavioral test was repeated twice with four different trials to validate the data.

The mNSS test consists of ten different tasks that can evaluate the motor (muscle status, abnormal movement), sensory (visual, tactile and proprioceptive), balance, and reflex functions of mice. Neurological function was graded from 0 to 18 (0 = normal function; 18 = maximal deficit). One point was scored for each abnormal behavior or for the lack of a tested reflex. Therefore, higher scores implying greater neurological injury.

The fine motor coordination and learning were assessed using an accelerating Rota-rod apparatus (RWD Life Science, Shenzhen, China). On the day before injury, mice were trained on the Rota-rod for three consecutive trials at a slow rotational speed (4 rpm/min) for 1 min to adapt to the rod, followed by four additional trials with an accelerating rotational speed (from 4 to 40 rpm in 5 min) to obtain baseline latency. On each testing day, the mice were given four 300-s accelerating Rota-rod trials with an inter-trial interval of 30 min. The average latency to the first fall off the rod was recorded. Passive rotation, accompanying the rotating rod without walking, was also considered as a fall.

### Flow cytometry analysis

For flow cytometry analysis of cellular components in the injured brain, the isolated cells were stained with fluorescently labeled antibodies: CD45-FITC, CD11b-APC, Ly6G-PE, CD3-PE-Cy7, CD45R (B220)-APC, NK1.1-PE, CD206-PE, CD86-PE-Cy7 and the appropriate isotype control according to the manufacturer’s protocols (eBioscience). To detect Tregs in the brain and spleen, the isolated cells were stained using a commercial Mouse Tregs Staining Kit (eBioscience). Briefly, cells were surface stained with anti-mouse CD4-FITC and anti-mouse CD25-PE for 30 min at 4 °C, fixed, and then permeabilized overnight at 4 °C using the fixation/permeabilization solution and subsequently stained with the anti-mouse/rat Foxp3-APC antibody. Flow cytometry was performed on a FACS Aria III apparatus (BD Bioscience) and the obtained data were analyzed by Flow Jo software 7.6.1(Tree Star, US).

### Immunohistochemistry staining

Paraffin-embedded sections were deparaffinized with gradient ethanol and xylene, and then boiled in a microwave with citrate buffer (pH 6.0) for 30 min to retrieve antigens. Following three washes with PBS, sections were incubated with 3% hydrogen peroxide and 3% bovine serum albumin (BSA, Sigma-Aldrich) to block endogenous peroxidase and nonspecific binding, respectively. The sections were then immunostained at 4 °C overnight with the respective primary antibodies: rabbit anti-Iba-1 antibody (1:500, Wako, Osaka, Japan), rabbit anti-MPO antibody (1:100, Abcam, Cambridge, UK) and, rabbit anti-CD3 antibody (1:100, Abcam). After primary antibody incubation, sections were incubated with biotinylated anti-rabbit IgG secondary antibody (1:500, Vector, Burlingame, CA, USA), and then overlaid with avidin–biotin horseradish peroxidase (HRP) complex (Vector). 3,3′-diaminobenzidine solution (DAB, Zsgb-bio, Beijing, China) was used to detect the HRP activity under light microscopy. The number of positive cells around the contusional cortex (see Fig. 1c) was calculated in six random microscopic fields (Olympus, Tokyo, Japan) of each section (three sections per animal) by ImageJ software (Version1.46r, Wayne Rasband, National Institute of Mental Health, USA) and the results were presented as the mean number of positive cells per square millimeter in the tiled images. All counts were performed in a blinded fashion.

### Immunofluorescence staining for neuronal apoptosis

To better assess neuronal apoptosis in the peri-contusional cortex, immunofluorescent double staining of terminal deoxynucleotidyl transferase-dUTP nick end labeling (TUNEL) and neuronal nuclei (NeuN) was conducted to determine colocalization of apoptotic cells and neurons. In brief, frozen sections were immunostained with mouse anti-NeuN antibody (1:100, Abcam) at 4 °C overnight and subsequently subjected to TUNEL staining using an in Situ Cell Death Detection kit (Roche, South San Francisco, CA, USA) according to the manufacturer’s suggested protocol. Finally, the sections were covered with 4′,6-diamidino-2-phenylindole (DAPI, Invitrogen). Positive cells around the injured cortex (see Fig. 1c) were calculated per square millimeter from six random microscopic fields of each section (three sections per animal) under a fluorescence microscope (Olympus). All counts were performed in a blinded fashion. The results were presented as the number of apoptotic neurons (NeuN-TUNEL double-stained cells) and the apoptotic ratio of the total neurons (NeuN-TUNEL double stained cells/NeuN-stained cells).

### Quantitative real-time polymerase chain reaction (qRT-PCR)

Total RNA of each brain sample was extracted using the TRIzol reagent (Invitrogen) according to the manufacturer’s protocol. The concentration of each RNA sample was quantified using ultraviolet spectrophotometry at 260/280 nm, only samples with an A260/280 > 1.8 were used for further analysis to ensure RNA quality. cDNA was transcribed using a SuperScript® III CellsDirect™ cDNA Synthesis Kit (Invitrogen) from 1.5 μg of RNA. qRT-PCR analysis was performed using an Opticon 2 Real-Time PCR Detection System (Bio-Rad, Hercules, CA, USA) with the SYBR® Green PCR Master Mix (Applied Biosystems, USA). The primers were subjected to 40 cycles of amplification at 95 °C for 10 s and 60 °C for 1 min. GAPDH was used as an internal control and the expression level of each target gene was normalized to that of GAPDH using the 2^−ΔΔCt^ method (Ct = threshold cycle).

The primer sequences were as follows:

MCP-1: forward 5′-GTGCTGACCCCAAGAGGAA-3′,

reverse 5′-TTGTGGAAAAGGTAGTGGATG C-3′

iNOS: forward 5′-TTGGAGCGAGTTGTGGATTG-3′,

reverse 5′-GTGAGGGCTTGGCTGAGTGA-3′

CD11b: forward 5′-CAGGGCAGGAGTCGTATTG-3′,

reverse 5′-GTCCATCAGCTTCGGTGTTG-3′

Arg-1: forward 5′-TGAACACGGCAGTGGCTTTA-3′,

reverse 5′-GTAGTCAGTCCCTGGCTTATGG-3′

YM1: forward 5′-GCAGAATAATGAGATCACTTACACAC-3′,

reverse 5′-ACGAAGGAATCTGATAACTGACTG-3′

CD206: forward 5′-AAACACAGACTGACCCTTCCC-3′,

reverse 5′- GTTAGTGTACCGCACCCTCC-3′

GAPDH: forward 5′- GGTGAAGGTCGGTGTGAACG-3′,

reverse 5′- CTCGCTCCTGGAAGATGGTG-3′

### Enzyme-linked immunosorbent assay (ELISA)

Total protein concentrations were measured using a BCA Protein Assay Kit (Thermo Fisher Scientific, Carlsbad, CA, USA). The levels of transforming growth factor-β1 (TGF-β1), interleukin-10 (IL-10), interferon-γ (IFN-γ), IL-6, regulated upon activation normal T-cell expressed and secreted (RANTES), and interferon-γ inducible protein-10 (IP-10) were measured using specific ELISA kits (Anoric-Bio, Tianjin, China) according to the manufacturer’s instructions.

### Statistical analysis

All the data were presented as the mean ± standard deviation (SD) and were analyzed using SPSS statistical software (version 22.0, IBM). Non-parametric data from the mNSS test were analyzed using the Kruskal-Wallis H analysis followed by a Mann-Whitney *U* test. One-way analysis of variance (ANOVA) with repeated measures with Bonferroni post hoc comparisons was used to analyze Rota-rod data. The remaining biochemical data were analyzed using a two-way ANOVA (TBI or sham) × (atorvastatin or saline) with post hoc Bonferroni multiple comparison test. The level of significance was set at a *P* value of <0.05.

## Results

### Atorvastatin treatment attenuates TBI-induced neurological deficits

To establish a dose-response for acute atorvastatin treatment post-TBI, we compared a range of dosing concentrations with vehicle saline following TBI. Prior to surgery, there were no differences among all the mice in both tests. In the mNSS test (Fig. 1d), mice in the sham groups showed mild neurological dysfunction at 24 and 72 h after craniotomy, while TBI caused significantly higher mNSS scores compared with the sham groups (*p* < 0.001). No significant differences were observed among the four TBI groups at 24 h post-TBI (*p* > 0.05). The mNSS scores decreased with time in all TBI groups because of spontaneous recovery. However, the scores were significantly lower in the 1, 5, and 10 mg/kg/day atorvastatin-treated groups compared with the saline-treated group at 72 h post-TBI (*p* < 0.01, 0.05, and 0.01, respectively). There was no significant difference among the three different dose groups at all tested time points. In the Rota-rod test (Fig. 1e), the sham groups exhibited the best performance compared with the other four TBI groups (all *p* < 0.001). At the same time, mice treated with 1, 5, and 10 mg/kg/day atorvastatin showed early improvement on the Rota-rod test, starting at 24 h post-TBI, when compared with that of the saline-treated mice, though no significant differences among the three atorvastatin-treated groups were found. Notably, we also observed that 1 mg/kg/day seemed more effective than higher 5 and 10 mg/kg/day, although the differences were not statistically significant.

The dose-response results demonstrated that no benefit of high doses (5 and 10 mg/kg/day) atorvastatin as compared to low dose (1 mg/kg/day) when the treatment was initiated 1 h after TBI, thus, a dose of 1 mg/kg/day was chosen for all subsequent experiments to minimize the possible side effects.

### Atorvastatin treatment alters brain-invading leukocyte subpopulations following TBI

**Fig. 2:**
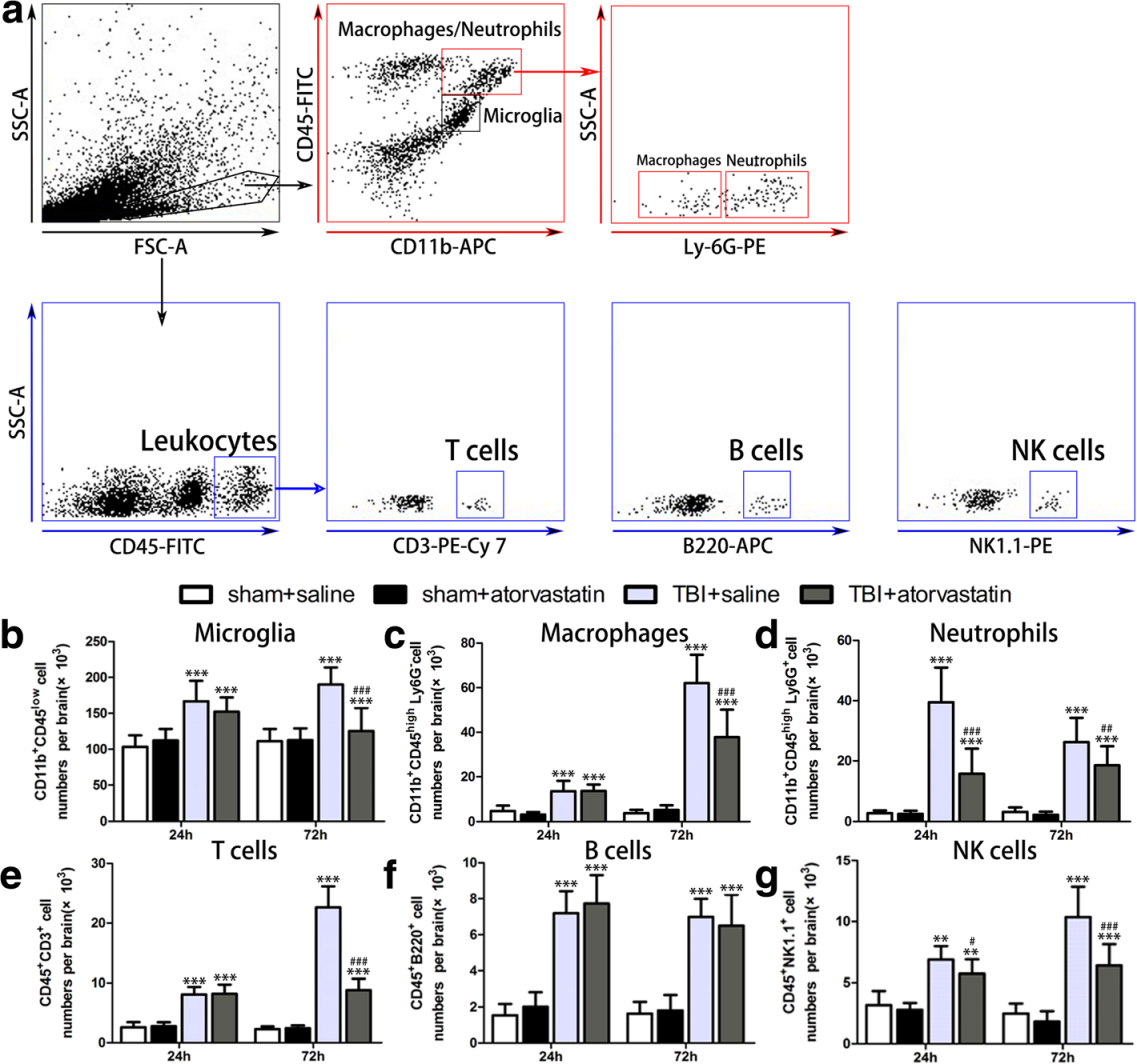
Effect of atorvastatin treatment on brain immune cell subsets after TBI. **a** Representative gating strategy of isolating microglia (CD11b^+^CD45^low^), macrophages (CD11b^+^CD45^high^Ly6G^−^), neutrophils (CD11b^+^CD45^high^Ly6G^+^), total T cells (CD45^+^CD3^+^), B cells (CD45^+^B220^+^), and NK cells (CD45^+^NK1.1^+^) infiltrating the brain. **b**–**g** Quantitative analysis of the invading cellular components in the different groups. Flow cytometric analysis showed a significant decline in microglia (**b**), macrophages (**c**), and T cells (**b**) in the atorvastatin group compared with the saline group at 72 h post-TBI. Atorvastatin administration had no effect on B cells (**f**) at 24 and 72 h following TBI. At 24 and 72 h post-TBI, atorvastatin treatment led to a significant reduction in the recruitment of neutrophils (**d**) and NK cells (**g**) to the injured brain when compared to the saline group. Data are presented as the mean ± SD. **p < 0.01 and ***p < 0.001 versus sham group, ^#^ *p* < 0.05, ^##^ *p* < 0.01, and ^###^ *p* < 0.001 versus TBI + saline group. *n* = 6/group. FSC-A = forward scatter channel area, SSC-A = side scatter channel area, FITC = fluorescein isothiocyanate, PE = phycoerythrin, and APC = allophycocyanin

**Fig. 3:**
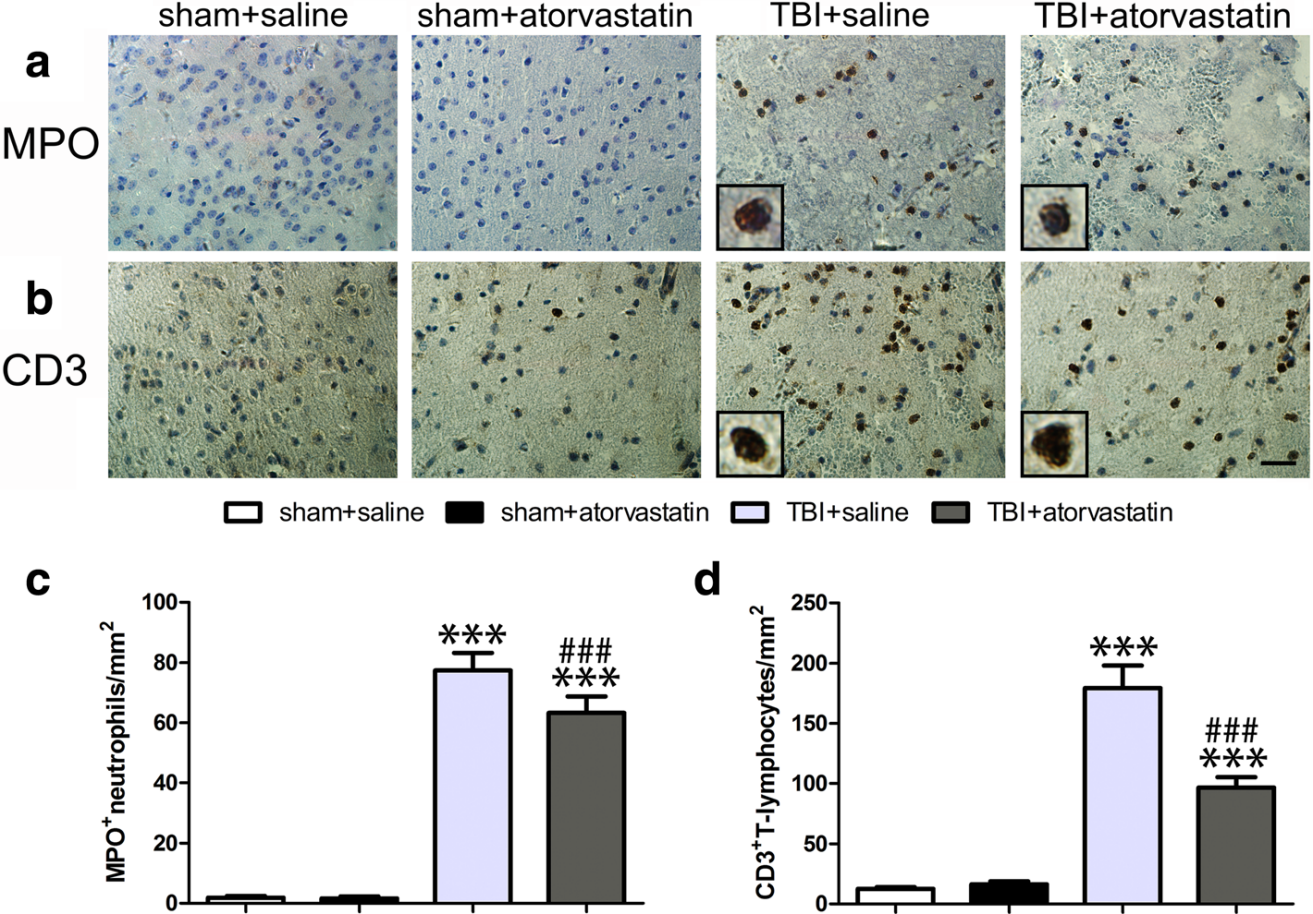
Effect of atorvastatin treatment on leukocyte invasion after TBI. **a**, **b** Representative immunohistochemically photomicrographs of neutrophils (MPO^+^, **a**) and T cells (CD3^+^, **b**) in the peri-contusional cortex after TBI. Scale bar = 200 μm. Inset display high magnification images of a positive cell. **c**, **d** Cell count analysis of neutrophils (**c**) and T cells (**d**) in the different groups. Atorvastatin-treated mice had significantly fewer infiltrated neutrophils (at 24 h), and infiltrated T cells (at 72 h) compared with those in saline-treated mice after TBI. Data are presented as the mean ± SD. ***p < 0.001 versus sham group, ^#^ *p* < 0.05 versus TBI + saline group. *n* = 6/group

To examine the effect of atorvastatin treatment on subpopulations of brain-invading leukocytes, quantitative and positioning analyses were assessed by flow cytometry and immunohistochemistry. In the flow cytometry analysis, the gating strategy is shown in Fig. 2a. We found that administration of atorvastatin reduced counts of T cells (CD45^+^CD3^+^) significantly in the brain at 72 h post-TBI when compared with the TBI + saline group (Fig. 2e, *p* < 0.001), whereas no changes in the invasion of B cells (CD45^+^B220^+^) were observed (Fig. 2f, *p* > 0.05). Moreover, significantly fewer neutrophils (CD11b^+^CD45^high^Ly6G^+^, Fig. 2d) and NK cells (CD45^+^NK1.1^+^, Fig. 2e) were detected in brain samples of the TBI + atorvastatin group at 24 and 72 h post-TBI compared with those in the TBI + saline group. Further immunohistochemical study of brain sections for MPO+ neutrophils and CD3+ T cells (Fig. 3) revealed that TBI induced a massive invasion of circulating neutrophils and T cells, and that these invading leukocytes were predominantly located in the contusional core and margin. Atorvastatin-treated TBI mice had significantly fewer neutrophils in the peri-contusional region at 24 h post-injury compared with those in the saline-treated TBI mice (Fig. 3c, *p* < 0.001). Furthermore, T cell invasion was also attenuated significantly by atorvastatin at 72 h post-injury (Fig. 3d, *p* < 0.001). In addition, no significant differences were observed between the two sham groups in all parameters. These results suggested that leukocyte infiltration profile was altered by atorvastatin treatment after TBI.

### Atorvastatin treatment increases Tregs in the spleen and brain following TBI

**Fig. 4:**
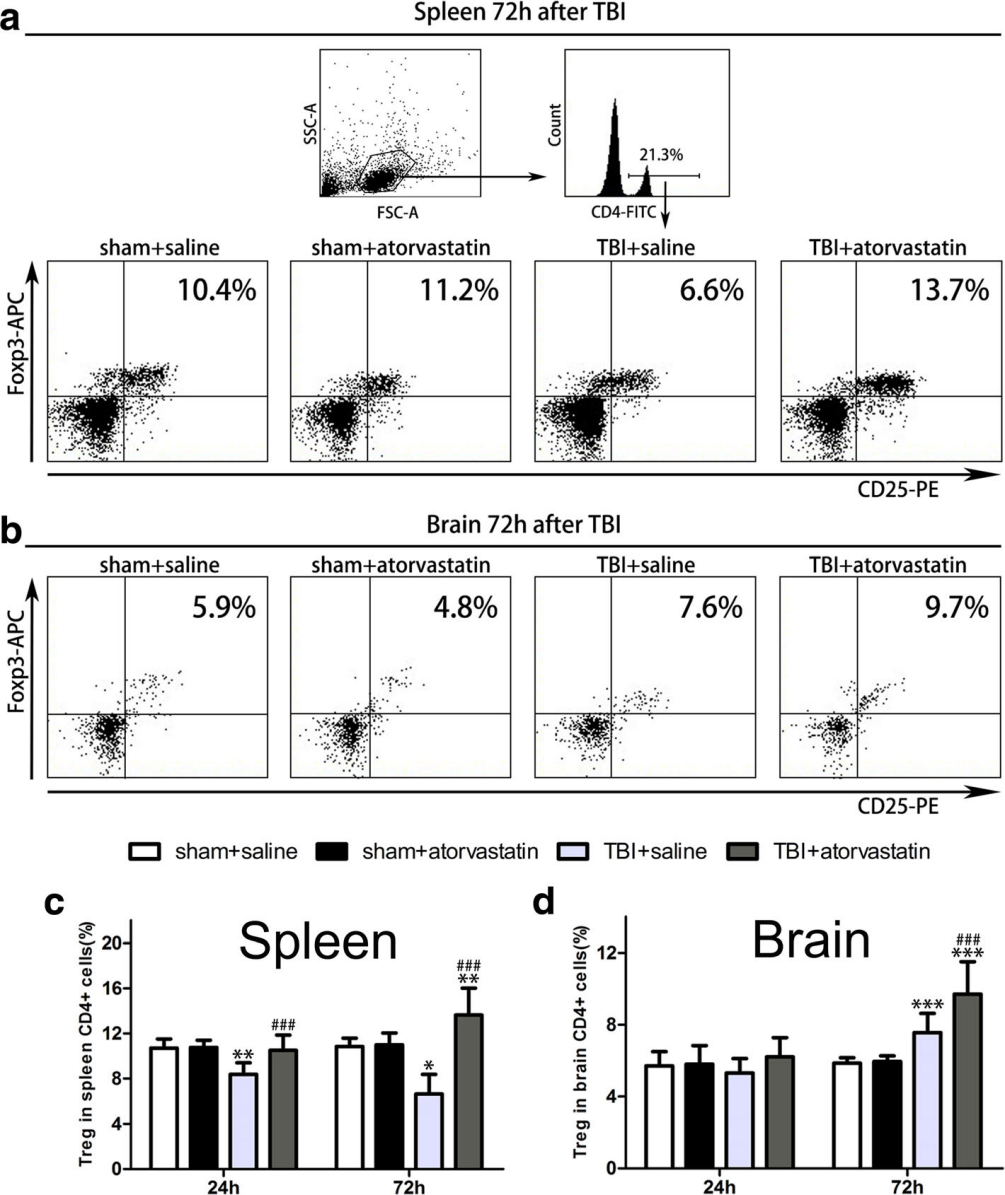
Effect of atorvastatin treatment on Tregs in the spleen and brain after TBI. **a, b** Representative dot plots showing the gating strategy of CD4 + CD25 + Foxp3 + Tregs from the peripheral spleen (**a**) and brain (**b**). Data are expressed as the Tregs in CD4+ T cells (%). **c, d** Quantitative analysis of the Tregs in the spleen and brain in the different groups. Mice in the TBI + saline group showed a decreased percentage of CD25 + Foxp3+ Tregs in spleen CD4+ T cells at 24 and 72 h (**c**); however, an increased percentage of Tregs in CD4+ T cells were present in the brain (**d**) at 72 h post-injury compared with mice in the sham groups. Atorvastatin treatment post-TBI increased the proportions of CD25 + Foxp3+ Tregs among CD4+ T cells significantly in both the spleen (**c**) and brain (at 72 h, **d**), compared with the TBI + saline group. Data are presented as the mean ± SD. *p < 0.05, **P < 0.01, and ***P < 0.001 versus sham group, ^###^ *P* < 0.001 versus TBI + saline group. *n* = 6/group. FSC-A = forward scatter channel area, SSC-A = side scatter channel area, FITC = fluorescein isothiocyanate, PE = phycoerythrin, and APC = allophycocyanin

To evaluate the effect of atorvastatin on Tregs after TBI, the levels of CD4 + CD25 + Foxp3+ Tregs from the peripheral spleen and the injured brain were analyzed by flow cytometry. The gating strategy is shown in Fig. 4a. Compared with the sham groups, the TBI + saline group showed a lower percentage of CD25 + Foxp3+ Tregs in the spleen CD4+ T cells at 24 (*p* < 0.01) and 72 h (*p* < 0.05) post-injury (Fig. 4c), but a higher level of Tregs in the brain at 72 h (Fig. 4d, *p* < 0.001), suggesting that TBI per se may upregulate the migration of peripheral Tregs to the injured brain. However, a significantly higher level of Tregs in the spleen and brain was observed in the atorvastatin group compared with that in the saline group at 24 and 72 h post-TBI. As expected, no difference was observed between the two sham groups. These results indicated that atorvastatin could expand CD4+CD25+Foxp3+ Tregs in both the peripheral spleen and the injured brain follow TBI.

### Atorvastatin treatment alters cytokine levels in peri-contusional cortex following TBI

**Fig. 5:**
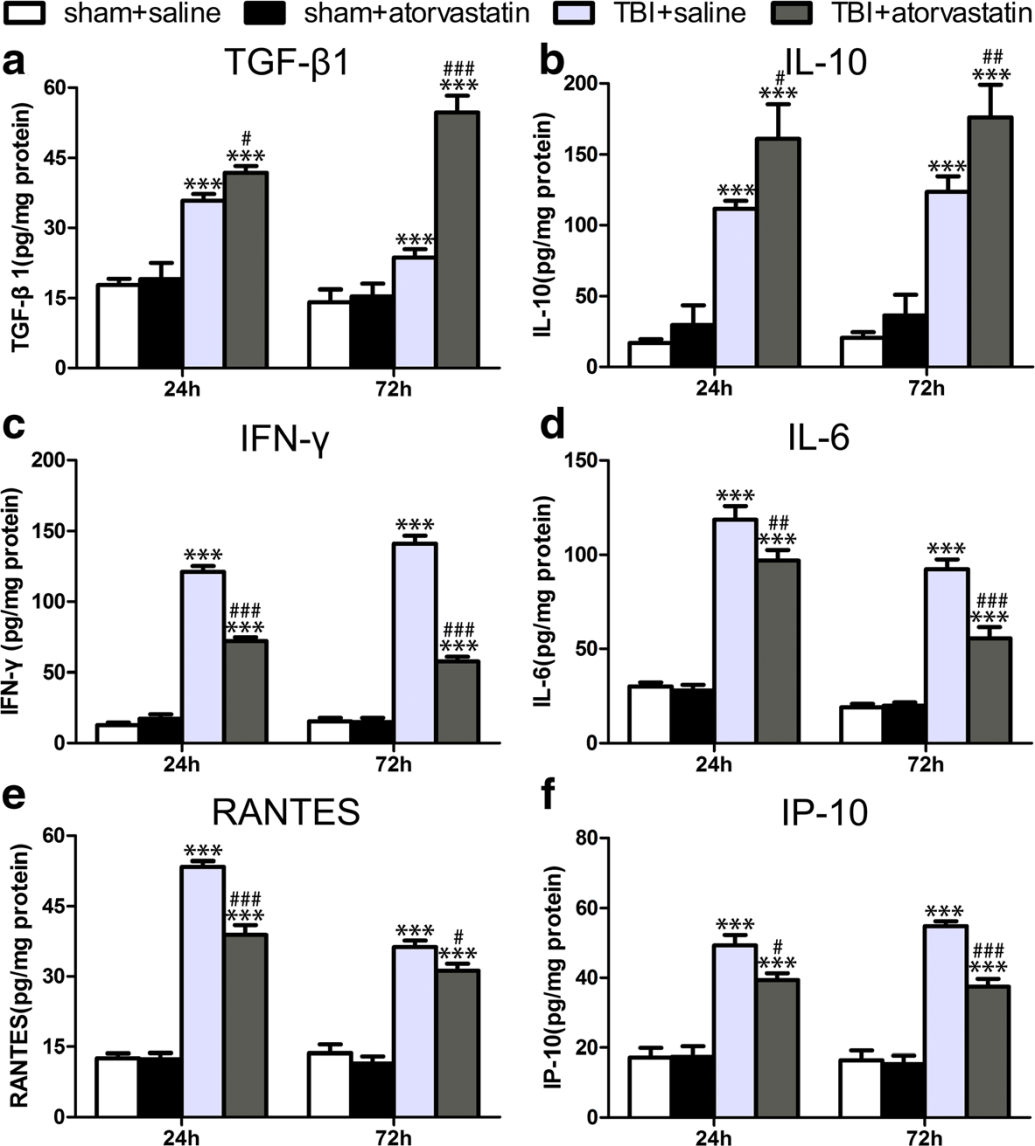
Effect of atorvastatin treatment on inflammatory cytokines expression in peri-contusional cortex after TBI. **a**–**d** Compared with the TBI + saline group, atorvastatin treatment increased the concentrations of the anti-inflammatory cytokines TGF-β1 (**a**) and IL-10 (**b**) significantly, whereas it reduced the levels of pro-inflammatory cytokines IFN-γ (**c**) and IL-6 (**d**) in the contusional boundary at 24 and 72 h after TBI. e-f The chemokines RANTES (**e**) and IP-10 (**f**) were also reduced at 24 and 72 h after TBI in the atorvastatin group compared with the TBI + saline group. Data are presented as the mean ± SD. ***P < 0.001 versus sham group, ^#^ *p* < 0.05, ^##^ *p* < 0.01, and ^###^ *P* < 0.001 versus TBI + saline group. *n* = 6/group

To determine the effect of atorvastatin on the expressions of inflammatory-associated mediators in the peri-contusional cortex, an array of inflammatory cytokines were measured using ELISA. The expressions of all detected cytokines were low in the sham groups. Protein levels of the anti-inflammatory cytokines TGF-β1 at 24 (*p* < 0.05) and 72 h (*p* < 0.001), IL-10 at 24 (*p* < 0.05) and 72 h (*p* < 0.01) post-TBI, increased in the TBI + atorvastatin group compared with those in the TBI + saline group (Fig. 5a, b). In contrast, levels of the pro-inflammatory cytokines IFN-γ at 24 (*p* < 0.001) and 72 h (*p* < 0.001), and IL-6 at 24 (*p* < 0.01) and 72 h (*p* < 0.001) post-TBI, all decreased significantly in the TBI + atorvastatin group compared with those in the TBI + saline group (Fig. 5c, d). The chemokines RANTES at 24 (*p* < 0.001) and 72 h (*p* < 0.05), and IP-10 at 24 (*p* < 0.05) and 72 h (*p* < 0.001) post-TBI, were reduced in atorvastatin group compared with those in the saline group (Fig. 5e, f). Notably, the expressions of all these inflammatory mediators in the sham group were not affected by atorvastatin.

### Atorvastatin treatment alleviates microglia/macrophage activation following TBI

**Fig. 6:**
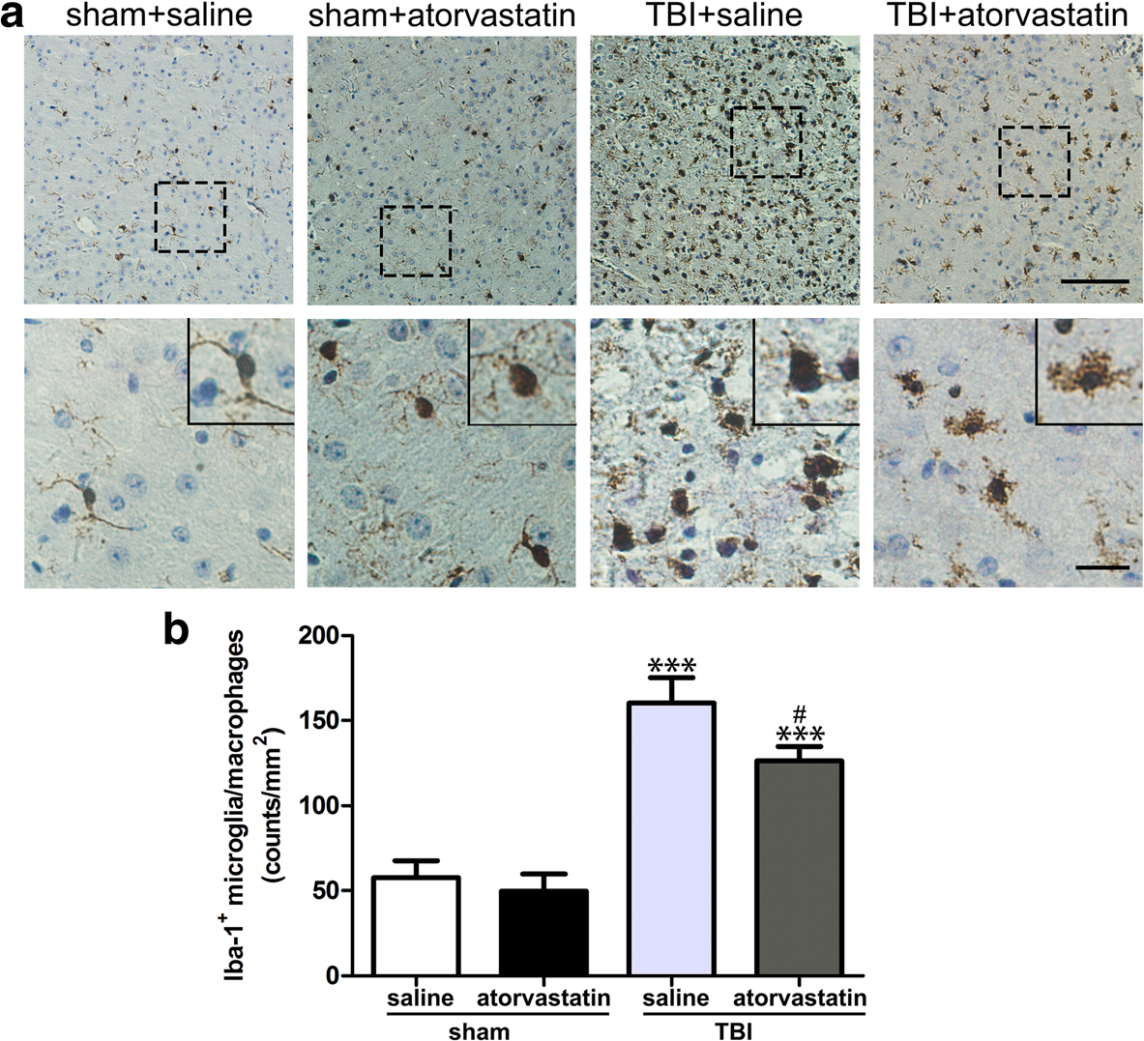
Effect of atorvastatin treatment on microglia/macrophage activation after TBI. **a** Representative immunohistochemical photomicrographs of Iba-1 stained microglia/macrophages in the peri-contusional cortex after TBI. Scale bar = 200 μm (upper,) and 100 μm (lower). Inset display high magnification images of a positive cell. Morphological observation showed that TBI provoked a drastic change in the morphology of microglia from the surveillant and ramified shape to a round and enlarged appearance. At 72 h after TBI, treatment with atorvastatin significantly reduced the soma size and ramification index. b Quantification of Iba-1 positive cells in the different groups. Atorvastatin-treated mice had significantly fewer activated microglia/macrophages compared with those in saline-treated mice at 72 h after TBI. Data are presented as the mean ± SD. ***p < 0.001 versus sham group, ^#^ *p* < 0.05 versus TBI + saline group. *n* = 6/group

To determine whether acute atorvastatin treatment could affect the microglia/macrophage activation, activated microglia or macrophages were detected by flow cytometry and immunohistochemistry. As shown in Fig. 2a, flow cytometry based gating study was performed to discriminate between brain-intrinsic microglia and infiltrating macrophages. At 72 h post-injury, there was a significantly reduced number of CD11b + CD45low microglia in the injured brain of atorvastatin-treated TBI mice when compared to saline-treated TBI mice (Fig. 2b, *p* < 0.001). Further analysis with a Ly6G antibody demonstrated that atorvastatin diminished mobilization of CD11b+CD45highLy6G- macrophages post-TBI (Fig. 2c, *p* < 0.001). Moreover, Iba-1 immunohistochemistry was performed to label total activated microglia/macrophages in the injured brain. As shown in Fig. 6a, the Iba-1+ cells in the sham groups were highly ramified, exhibiting small cell bodies and high degree of ramifications. In contrast, TBI provoked a drastic change from the ramified shape to larger cell bodies with fewer ramifications (hypertrophic or amoeboid shape). Compared with the saline-treated mice, the atorvastatin-treated mice showed smaller soma size and higher ramification index, and had significantly fewer Iba-1+ cells in the peri-contusional region (Fig. 6b, *p* < 0.05) at 72 h post-TBI. These results suggested that atorvastatin treatment not only decreased total activated microglial/macrophage numbers but also induced a morphological shift from the amoeboid shape toward the surveillant ramified morphology.

### Atorvastatin treatment alters M1/M2 microglia/macrophage polarization following TBI

**Fig. 7:**
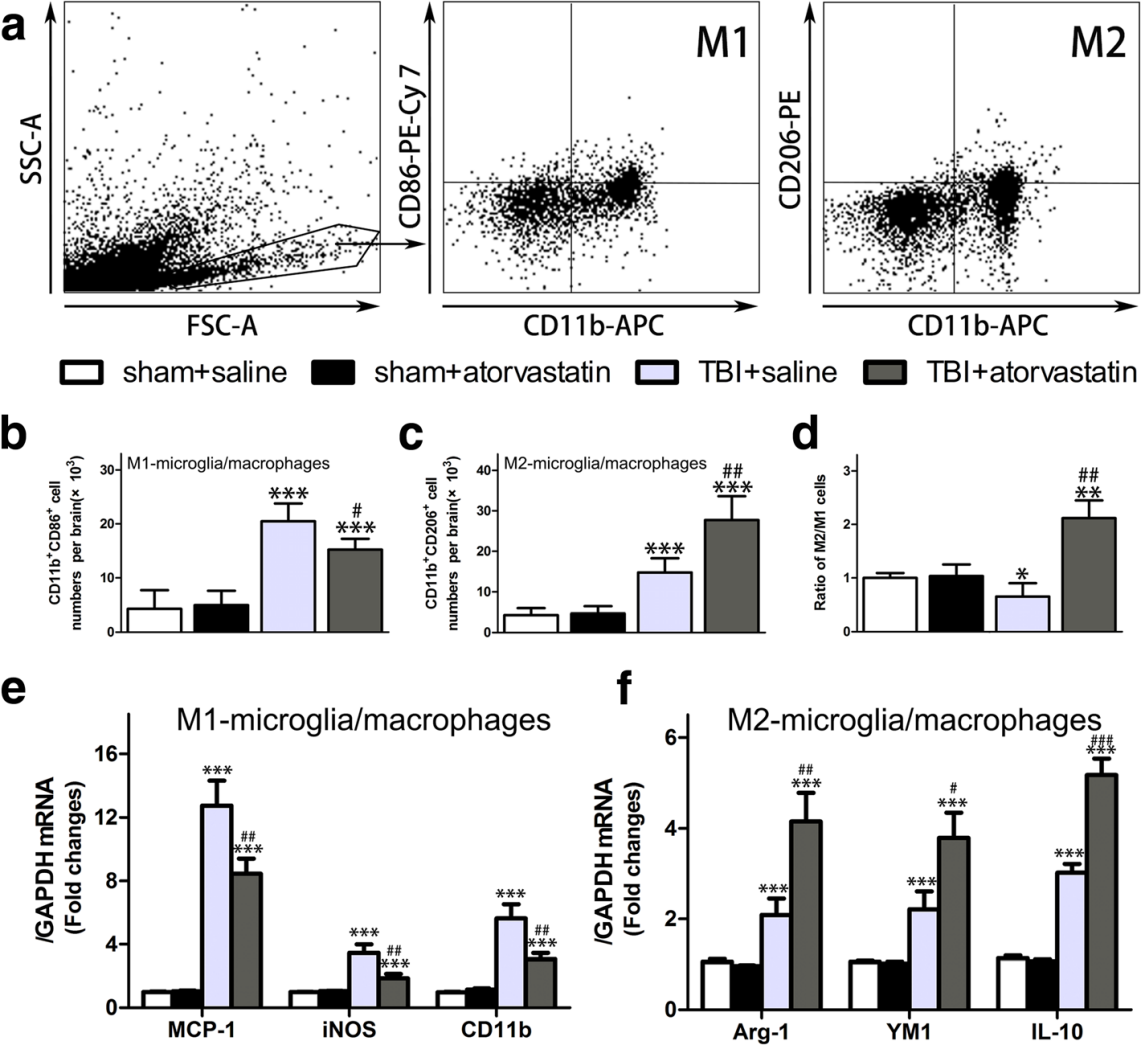
Effects of atorvastatin treatment on M1/M2 microglia/macrophage polarization after TBI. **a** Representative gating strategy of M1 microglia/macrophages (CD11b^+^CD86^+^), and M2 microglia/macrophages (CD11b^+^CD206^+^). **b**–**d** Quantitative analysis of the M1, M2 cells and the ratio of M2/M1 in the different groups. Atorvastatin-treated mice had significantly fewer M1 cells, more M2 cells, and higher M2/M1 ratio compared with saline-treated mice at at 72 h after TBI. e qRT-PCR results for the M1-type mRNA expression of MCP-1, iNOS and CD11b. f M2-type mRNA expression of Arg1, Ym1/2 and CD206. Expression levels of the mRNAs were normalized to that in the sham control. TBI induced a marked increase in both M1- and M2-type mRNA expression in the injured brains of mice compared with the sham groups. However, atorvastatin administration significantly attenuated M1 related gene expressions and promoted M2 related gene expressions compared with those in the TBI + saline group at 72 h post-injury. Data are presented as the mean ± SD. **P < 0.01 and ***p < 0.001 versus sham group, ^#^ *p* < 0.01, ^##^ *p* < 0.01, and ^###^ *P* < 0.001 versus TBI + saline group. *n* = 6/group

We further investigate microglial/macrophage activation by examining their polarization status after TBI. As shown in Fig. 7a, CD11b-positive microglial/macrophages were labeled with markers for M1 (CD86) and M2 (CD206) phenotypes. Flow cytometric analysis clearly demonstrated that atorvastatin treatment would reduce pro-inflammatory M1 cells (CD11b^+^CD86^+^, Fig. 7b, *p* < 0.05) and increase anti-inflammatory M2 cells (CD11b^+^ CD206^+^ Fig. 7c, *p* < 0.01), thereby augmenting M2/M1 ratio (Fig. 7d, *p* < 0.01) at 72 h post-TBI compared with that in the TBI + saline group. In addition, gene expression related to the M1/M2 phenotype in the peri-contusional region of brain tissues was analyzed using qRT-PCR assay. Consistent with the flow cytometry results, TBI induced dramatic increases in the expression of all M1/M2 markers compared with those in the sham groups at 72 h post-injury (Fig. 7e, f). With the administration of atorvastatin, the mRNA expression levels of M1-type genes MCP-1, iNOS, and CD11b were decreased significantly, whereas the expressions of all the tested M2-type gene markers, including Arg1, Ym1, and CD206, increased dramatically compared with those in the TBI + saline group. Taken together, these results demonstrated that acute atorvastatin treatment could significantly alter the M1/M2 phenotype balance, via both inhibiting M1 activation and enhancing M2 activation after TBI.

### Atorvastatin treatment attenuates TBI-induced neuronal apoptosis

**Fig. 8:**
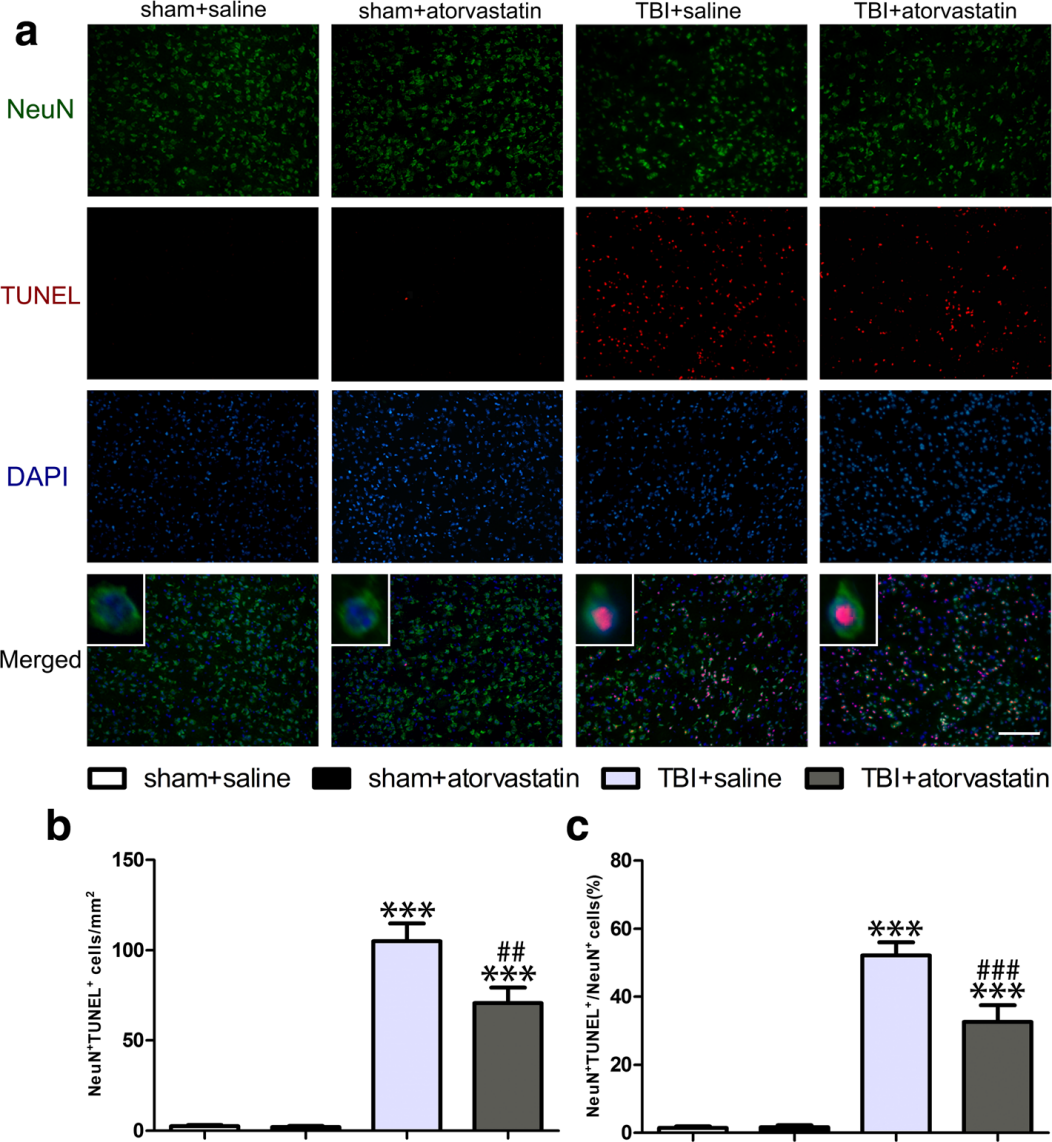
Effect of atorvastatin treatment on neuronal apoptosis after TBI. **a** Representative fluorescence images of TUNEL-positive neurons in the peri-contusional cortex at 72 h after TBI. Fluorescence colors: TUNEL: red, NeuN: green, and DAPI: blue. Scale bar = 200 μm. TUNEL and NeuN double stained cells indicated the apoptotic neurons, overlapped images showed that TUNEL-positive cells mainly colocalized with neurons. **b** Quantitative analysis of apoptotic neurons in the different groups. Few TUNEL-positive apoptotic neurons were detected in the sham groups. Apoptotic neurons in the peri-contusional cortex at 72 h post-TBI were reduced in the atorvastatin treatment group compared with those in the saline group. **c** The ratio of apoptotic neurons in total neurons. Compared with the TBI + saline group, administration of atorvastatin significantly decreased the apoptosis ratio. Data are presented as the mean ± SD. ***p < 0.001 versus sham group, ^##^ *p* < 0.01 and ^###^ *P* < 0.001 versus TBI + saline group. *n* = 6/group

As depicted in Fig. 8a, double staining of TUNEL and NeuN showed that TUNEL-positive apoptotic cells mainly occurred in neurons. There were almost no TUNEL-positive neurons in the right cortex in the sham groups, and the number of apoptotic neurons in the peri-contusional cortex increased significantly in the TBI groups compared with that in the sham groups (*p* < 0.001). However, compared with the TBI + saline group, treatment with atorvastatin substantially reduced the number of apoptotic neurons at 72 h post-TBI (Fig. 8b, *p* < 0.01). Furthermore, the percentage of apoptotic neurons in total NeuN-positive neurons was also analyzed. As shown in Fig. 8c, a larger percentage of apoptotic neurons in TBI groups was observed (*p* < 0.001), whereas atorvastatin promoted neuronal survival by attenuating the percentage of apoptotic neurons (*p* < 0.001).

## Discussion

In the present study, we investigated the anti-inflammatory and immunomodulatory properties of atorvastatin in mice post-TBI. The main findings were that acute (1 h post-trauma) atorvastatin treatment significantly reduced neuronal damage and improved short-term functional recovery. Atorvastatin altered the recruitment of peripheral leukocyte subsets at the injured site, as well as decreasing pro-inflammatory cytokines and chemokines, and increasing anti-inflammatory cytokines. We also observed that acute administration of atorvastatin attenuated the often-destructive M1 microglia/macrophage phenotype, while simultaneously increasing the M2 phenotype post-TBI. Based on the well patient tolerance to statins and their ease of delivery, the present data provides us with a compelling rationale to translate such work into clinical studies.

There have been numerous pre-clinical studies focusing on the neuroprotective effects of oral administration of statins after acute brain injury. As most early studies stated, when the treatment was initiated in hours post-injury, statins at high doses of 10 mg/kg/day or more were necessary to exert robust neuroprotective effects in stroke and TBI [24, 27, 28]. However, when initial treatment was delayed (24 h post-injury), low-dose statins, ranging from 1 to 3 mg/kg/day, had beneficial roles including increasing angiogenesis, neurogenesis, and functional neurological outcomes. Interestingly, a higher dose of 8 mg/kg/day was ineffective or even deleterious [29–31]. In addition, still other researchers demonstrated that statins had no functional benefit in TBI [32]. These mixed results may be partly attributed to a number of variables, including optimal statin type, dosage (1 to 100 mg/kg/day), duration (3 to 14 days), and the time-point of initial treatment (1, 3, 6, or 24 h post-injury). To date there are scant data regarding the effect of early initiation of atorvastatin treatment during the acute phase of TBI. Therefore, before studying the anti-inflammatory and immunomodulatory effects, we performed a dose-effect study for atorvastatin, administered 1 h after injury, on post-TBI sensory-motor deficits (assessed by mNSS) and motor dysfunctions (assessed by Rota-rod). We found that acute atorvastatin treatment significantly reduced functional deficits as early as 24 h post-TBI. Notably, we also observed that 1 mg/kg/day atorvastatin seemed more effective than higher 5 and 10 mg/kg/day atorvastatin, although the differences were not statistically significant. These data were in agreement with a recent study of rosuvastatin, which reported that no benefit of 5 mg/kg/day rosuvastatin as compared to 1 mg/kg/day when administered within 1 h post-TBI [26]. Considering the possible higher incidence of adverse effects when translated to clinical practice using high dose, we chose 1 mg/kg/day for the further study.

While the role of T cells in mediating inflammatory neuropathology has been studied intensively, their exact role in TBI remains a matter of debate. Studies suggest that T cells are crucial detrimental modulators that promote CNS inflammation and exacerbate neuronal injury [33, 34]. Moreover, lymphocyte-deficient (Rag1−/−) mice exhibited profound protection against acute neuroinflammation in a cerebral freeze-injury model [14]. While Weckbach et al. reported that no difference between Rag1−/− and wild-type mice in closed head injury. The discrepancy might be explained by the magnitude of immune cell invasion according to the distinct experimental model systems [35]. In addition, T cells also have beneficial effects on the CNS repair in the chronic phase post-injury [10]. Hence, prevention of excessive T cells invasion at the acute phase of TBI might be a promising strategy to promote tissue recovery. In the present study, acute atorvastatin treatment reduced the invasion of T cells into the CNS. Interestingly, we also found that atorvastatin increased the Tregs proportion significantly in both the peripheral spleen and brain CD4+ T cells. Tregs are an immunosuppressive subtype of T cells that have the capability to limit the overactivated immune system and dampen inflammation post-injury [36]. For instance, they can suppress the function of effector T cells and the activation of monocytes/macrophages through either cell-cell interactions or by the production of anti-inflammatory cytokines TGF-β and IL-10 [37]. The level of circulating Tregs was significantly lower in TBI patients with poor prognosis, suggesting that increasing Tregs might improve the clinical outcome after TBI [38]. Our results were consistent with a previous study that indicated that atorvastatin could modulate Tregs in peripheral tissue and favor their accumulation in the brain after ischemic stroke [24].

Additionally, NK cells, a type of cytotoxic lymphocytes, are increasingly recognized as prominent effectors in the adaptive immune response [39]. NK cells infiltrate the ischemic hemispheres during the acute phases of cerebral ischemia, which contribute to early inflammation and neuronal damage via IFN-γ [40–42]. Moreover, administration of NK cells antagonists led to a significant reduction of infarct size in mice subjected to permanent middle cerebral artery occlusion [42]. However, data on the influence of NK cells during TBI is scarce. Kong et al. proved that the level of circulating NK cells correlated positively with clinical outcomes after TBI [43]. Here, we indicated that NK cells were trafficked into the injured brain as early as 24 h post-TBI, and atorvastatin treatment dramatically reduced their invasion. However, additional research is required to clarify the underlying mechanisms of these phenomena. In addition, the exact role of NK cells in TBI, including the temporal profile of their infiltration, requires further study.

Cytokines and chemokines play a pivotal role in CNS inflammation by regulating leukocyte activation, expansion and migration toward the injured CNS [44, 45]. RANTES, also known as C-C motif chemokine ligand 5 (CCL5), contributes to T cell and monocyte infiltration and subsequent secondary brain damage following TBI. Laboratory and human studies have shown upregulation of RANTES mRNA in the brain tissue after TBI [46]. Furthermore, higher circulating RANTES levels in TBI patients are associated positively with poorer clinical outcomes [47]. IP-10, also known as C-X-C motif chemokine ligand 10 (CXCL10), is also a chemoattractant for T cells that contributes to brain damage [48]. In spinal cord injury, Liu G et al. found that increased expression of CCL5 and CXCL10 preceded or paralleled T cell invasion, and both could also induce T-cell proliferation and cytokine production [49]. In addition, IP-10 is also a primary chemotactic factor for NK cells infiltrating the CNS [42]. IFN-γ is a pleiotropic cytokine that is mainly released by invading T cells and NK cells, and can aggravate neuroinflammation by inducing further production of pro-inflammatory cytokines and ROS [50]. May et al. reported that IFN-γ could upregulate the expression of leukocyte adhesion molecules, thus promoting the transmigration of leukocytes across the endothelium [51]. However, the other two cytokines, IL-10 and TGF-β1, secreted by multiple cells including Tregs, have the ability to ameliorate TBI-induced inflammatory damage by suppressing microglia and T cells activation, pro-inflammatory cytokines production, and peripheral leukocyte recruitment [52, 53]. The present results showed that atorvastatin treatment suppressed IFN-γ, IL-6, RANTES, and IP-10 expression, while increasing IL-10 and TGF-β1 expression. The oppositely modulated production of pro- and anti-inflammatory cytokines and chemokines might partially explain the atorvastatin-induced decrease in the recruitment of blood-derived leukocytes.

Activated microglia/macrophages are the primary executors in the process of CNS inflammation, and can be polarized into a detrimental phenotype (M1) or a beneficial phenotype (M2) according to their host tissue microenvironments [17]. A shift to beneficial M2 microglia/macrophages can suppress neuroinflammation and promote CNS repair [19]. However, the TBI-induced vulnerable brain microenvironment changes a transient M2 phenotype to a sustained M1 phenotype in both white and gray matter [54, 55]. In the present study, we found that acute atorvastatin treatment strongly inhibited the activation and recruitment of microglia and brain-infiltrating macrophages, shifting their morphology back into a ramified state, and altered the polarization of microglia/macrophages by inhibiting M1 activation and promoting M2 activation. One possible explanation is atorvastatin’s effect on the modulating cytokine-dependent tissue microenvironment [54, 56]. Furthermore, Tregs were reported to inhibit microglia/macrophage activation and shift the polarization of microglia/macrophages toward the protective M2 phenotype, while CD4 + CD25- T cells displayed the opposite effects by promoting M1 polarization [15, 57–59]. Taken together, we postulated that atorvastatin could regulate the status of microglia/macrophage polarization toward M2 phenotype via modulating lymphocyte infiltration and altering the expressions of pro- and anti-inflammatory cytokines.

## Conclusions

We demonstrated that atorvastatin provides significant neuroprotection against TBI via anti-inflammatory and immunomodulatory effects, which resulted from altering the invasion of peripheral leukocyte subsets and the polarization status of microglia/macrophages. These findings extend the current understanding of the mechanisms regarding the protective effects of atorvastatin following TBI. Furthermore, our findings suggest that neuroinflammation-targeted therapy post-TBI should aim at reasonable modulation, rather than overall elimination, of the inflammatory response.

## Abbreviations

ANOVA: One-way analysis of variance
APC: Allophycocyanin
APC: Antigen presenting cells
BBB: Blood–brain barrier
BSA: Bovine serum albumin
CCI: Controlled cortical impact
CCL5: C-C motif chemokine ligand 5
CNS: Central nervous system
CXCL10: C-X-C motif chemokine ligand 10
DAB: 3,3′-diaminobenzidine solution
DAPI: 4′,6-diamidino-2-phenylindole
ELISA: Enzyme-linked immunosorbent assay
FITC: Fuorescein isothiocyanate
HMG-CoA: 3-hydroxy-3-methyl-glutaryl-coenzyme A
HRP: Avidin–biotin horseradish peroxidase
IFN: Interferon
IL: Interleukin
IP: Interferon-γ inducible protein
mNSS: Modified neurological severity score
NeuN: Neuronal nuclei
NK: Natural killer
PBS: Phosphate-buffered saline
PE: Phycoerythrin
qRT-PCR: Quantitative real-time polymerase chain reaction
RANTES: Regulated upon activation normal T-cell expressed and secreted
ROS: Reactive oxygen species
TBI: Traumatic brain injury
TGF: Transforming growth factor
Tregs: Regulatory T cells
TUNEL: Terminal deoxynucleotidyl transferase-dUTP nick end labeling
